# The shallow structure of Solfatara Volcano, Italy, revealed by dense, wide-aperture seismic profiling

**DOI:** 10.1038/s41598-017-17589-3

**Published:** 2017-12-12

**Authors:** Pier Paolo G. Bruno, Stefano Maraio, Gaetano Festa

**Affiliations:** 1 Khalifa University of Science and Technology, Petroleum Institute, P.O. Box 2533 Abu Dhabi, United Arab Emirates; 2 Università degli Studi di Siena, Centro di GeoTecnologie, San Giovanni Valdarno, Italy; 3 Università degli studi di Napoli Federico II, Dipartimento di Fisica “Ettore Pancini”, Naples, Italy

## Abstract

Two active-source, high-resolution seismic profiles were acquired in the Solfatara tuff cone in May and November 2014, with dense, wide-aperture arrays. Common Receiver Surface processing was crucial in improving signal-to-noise ratio and reflector continuity. These surveys provide, for the first time, high-resolution seismic images of the Solfatara crater, depicting a ~400 m deep asymmetrical crater filled by volcanoclastic sediments and rocks and carved within an overall non-reflective pre-eruptive basement showing features consistent with the emplacement of shallow intrusive bodies. Seismic reflection data were interpreted using the trace complex attributes and clearly display several steep and segmented collapse faults, generally having normal kinematics and dipping toward the crater centre. Fault/fracture planes are imaged as sudden amplitude drops that generate narrow low-similarity and high-dip attributes. Uprising fluids degassed by a magmatic source are the most probable cause of the small-scale amplitude reduction. Seismic data also support the interpretation of the shallow structure of the Solfatara crater as a maar. Our results provides a solid framework to constrain the near-surface geological interpretation of such a complex area, which improves our understanding of the temporal changes of the structure in relation with other geophysical and geochemical measurements.

## Introduction

**Figure 1:**
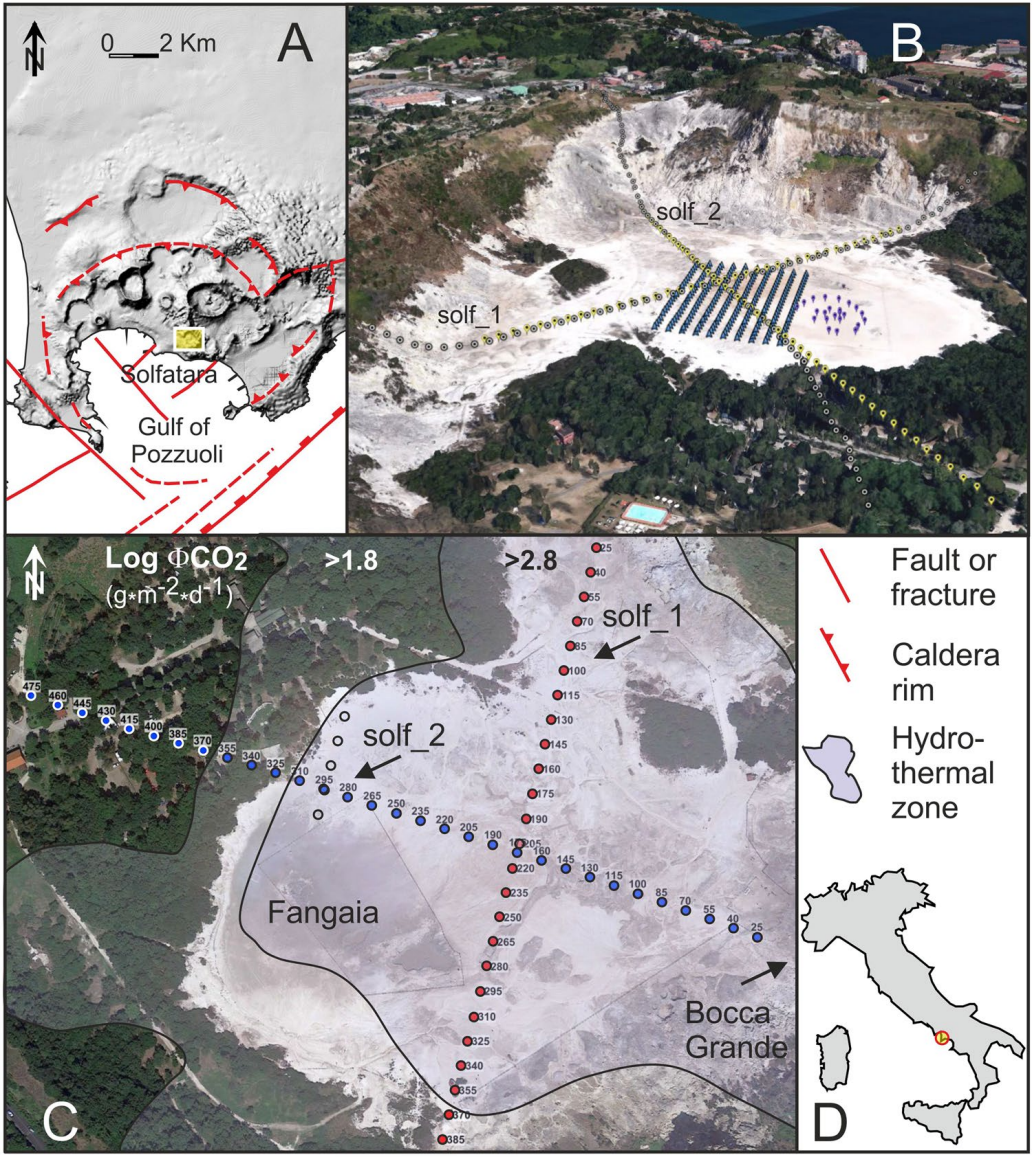
(**A**) DEM map of Campi Flegrei Caldera with a sketch of the main volcanic and tectonic features^33,34^ modified from Bruno *et al*.^11^. The yellow box locates the Solfatara. (**B)** 3D Image map of Solfatara (© 2017 Google), showing the location of the MED-SUV RICEN arrays from a NW perspective. (**C**) 2D image map of Solfatara (© 2017 Google), showing the CDP locations of the 2D seismic arrays. Red dots: profile solf_1; blue dots: profile solf_2. The distribution of CO2 flux^7^ is overlapped to the image. The inset in the lower right (**D**) shows the location of the figure with respect to the Italian Peninsula.

Campi Flegrei (hereinafter named as CF, Fig. 1) is a large, 13-km-wide, nested caldera located within the metropolitan area of the city of Naples, Italy and formed by two main eruptive events with volcanic explosive indexes greater than five (Campanian Ignimbrite; ~39 ka and Neapolitan Yellow Tuff; ~15 ka). In particular, the ~39 ka Campanian Ignimbrite event, has been the largest eruption occurred in Europe during the last 200 ka^1^. Based on its volcanological history, its location and on the fact that calderas can erupt with little warning, preceded only by small unrest signals^2,3^, CF can be certainly regarded as one of areas at highest volcanic risk of the planet. The last CF eruption occurred in 1538 AD. Since the 1950s, CF has been showing clear signs of resurgence, as shown by important and recurring episodes of ground deformation^4^; by seismicity^5^ (only associated to uplift episodes) and by an evident increase of hydrothermal degassing^6^, which witness a change of state of the CF magmatic system that may ultimately culminate in rock failure and eruption^4,6^. Almost all recent (i.e. 2000–2016) CF earthquakes are clustered inside a 2.5 km radius circle centred at the Solfatara crater (Fig. 1), and above a depth of 2 km^5^. Solfatara crater is also the most hydrothermally active area of CF and releases ~1500 tons of CO_2_ and more than 3000 tons of water vapour every day^7^. Most of the water vapour discharge condenses near the surface, producing a thermal power flux of ~100 MW, and contributing notably to the total water input into the CF hydrothermal system^7^. Caliro *et al.* ^8^ suggest that deep magmatic CO_2_-rich fluids mix with liquids of meteoric origin and form a hydrothermal plume that feeds the fumaroles of the Solfatara. De Siena *et al*.^9^ found that the subsurface volume affected by this hydrothermal plume shows high seismic attenuation. Active-source seismic studies^10^ locate the deepest magmatic source of this hydrothermal system at ~7.5 km deep. Bruno *et al*.^11^ performed several geophysical surveys inside Solfatara and hydrogeological measurements in 30 boreholes in the area surrounding the crater, in order to depict the upper structure of the hydrothermal plume. The water table position clearly identifies the upwelling of the piezometric surface produced by the plume, with the apex below the Solfatara crater. Resistivity tomography surveys^12^, combined with mappings of diffuse CO_2_ flux, ground temperature and self-potential allowed delineating two shallow plume structures: a liquid-dominated conductive plume below the Fangaia mud-pool and a gas-dominated plume below Bocca Grande fumarole.

In this very complex environment, we conducted the RICEN (Repeated Induced Earthquakes and Noise) experiment, aimed at characterizing the shallow structure of the Solfatara and at monitoring its changes with time by performing repeated experiments in a 3D time-lapse configuration^13^. Using the active-source data from the 3D RICEN array (Fig. 1B,C) de Landro *et al*.^14^, obtained high-resolution tomographic P-wave velocity images of the first 30 m of the central part of the crater. These images show a large spatial heterogeneity, caused by poorly consolidated sediments affected by a different degree of water and/or CO_2_ saturation.

To image the structure of the crater at larger depths (down to ~1 km), during the RICEN experiment we also conducted two high-resolution seismic profiles along two orthogonal NNE (i.e. solf_1) and WNW (i.e. solf_2) directions (Fig. 1B,C). Their purpose is to locate fractures and faults, whose position and geometry are fundamental in understanding the mechanisms of massive degassing and hydrothermal fluid circulation occurring near the surface. In particular, for data processing and interpretation, we make use of the tools from high-resolution reflection seismology. However, hydrothermal conditions very often imply the occurrence of large subsurface heterogeneities, owing to the presence of gas and steam pockets, non-horizontal interfaces and high-temperature, altered rocks and sediments. These conditions make the processing challenging because such media strongly attenuate the high-frequency component of the signal. The basic assumptions of conventional Common Depth Point (hereinafter named as CDP) processing, i.e. horizontal reflectors and smooth lateral and vertical velocity gradients, are commonly violated, pushing the acquisition and the processing toward non-conventional approaches^15^. The two seismic profiles were acquired using dense-wide-aperture arrays^16^, which differ from a typical CDP reflection array since they record both dense high-fold reflection data spanning a wide range of offsets and deep penetrating refracted waves, suitable for first-arrival traveltime tomography. Moreover, dense wide-aperture allows for capturing, much better than short arrays, both reflected and refracted waves from dipping targets, such as fault planes and seismic interfaces with complex morphology. In settings where the high sensitivity to velocity analysis makes conventional seismic data processing unfeasible, seismic imaging can be achieved using the Common Reflection Surface (CRS) stacking, a data-driven and velocity-independent method^17^. The CRS stack can produce zero-offset sections with high signal-to-noise ratio, improved resolution, more continuous reflectors, and enhanced images of dipping reflectors^18^. In contrast to CDP stacking, the great advantage of CRS stack is that it does not directly depend on the velocity model.

## Results

**Figure 2:**
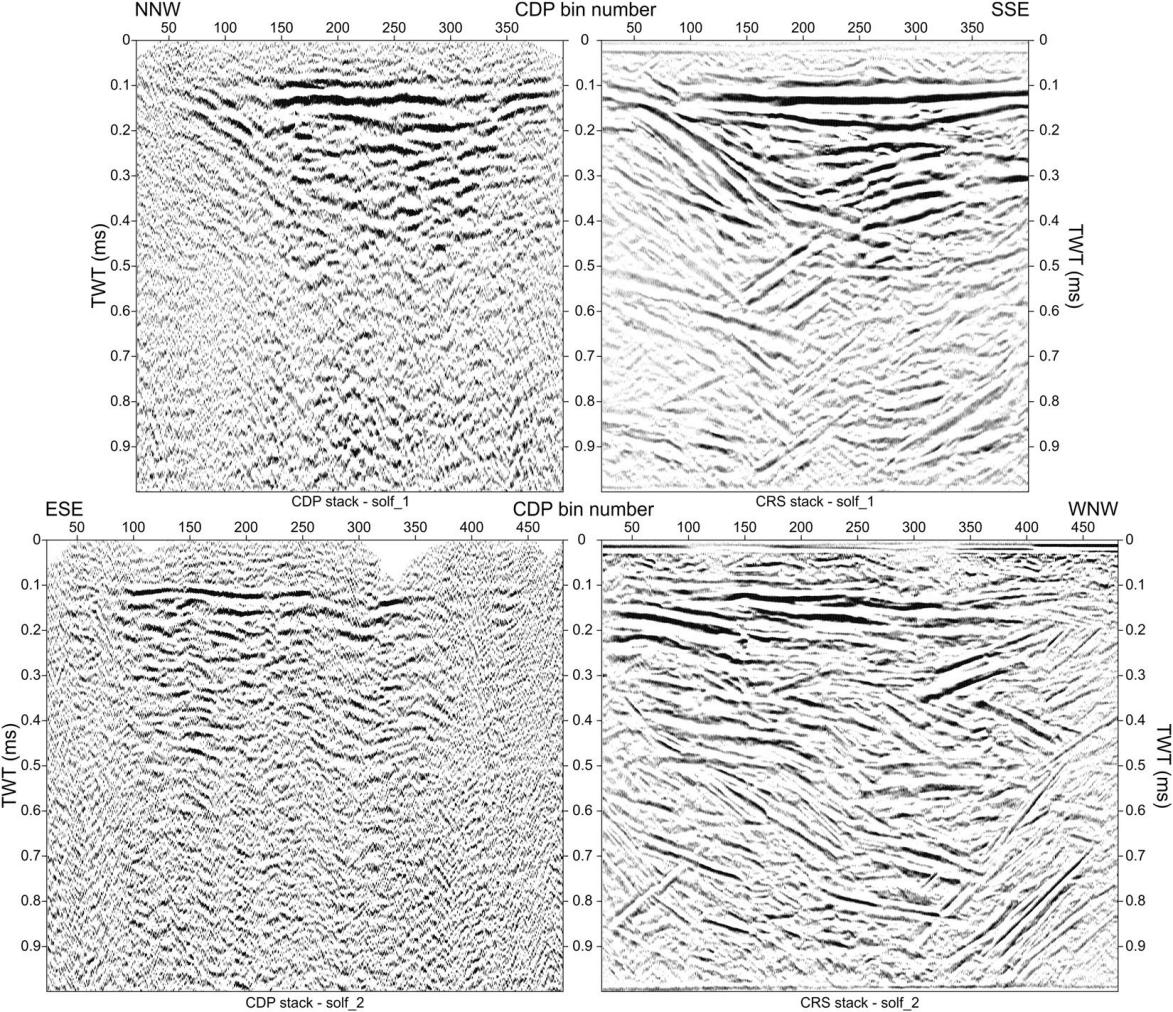
Comparison between the CMP stacks obtained for profile solf_1 (top) and solf_2 (bottom) through a standard CDP processing (left) and by using the CRS stack processing (right). Data are plotted with a horizontal exaggeration of ~2.

In Fig. 2 we compare the results of CDP and CRS stacking for both profiles acquired at Solfatara. Reflection profiles target the reflective volcanoclastic filling of the volcano, interpreted by Isaia *et al*.^19^ as a maar-diatreme structure. The crater bottom is visible approximately at ~0.5 s Two-Way-Time (TWT: i.e., the time from surface down to the reflector and back). CDP stacks exhibit low signal-to noise ratio and reflectivity down to ~0.5 s TWT. In particular, signal-to-noise ratio and penetration is lower for the CDP stack of solf_2. Comparison of CDP with CRS stacks reveals that CRS has dramatically improved the signal-to-noise ratio and the reflector continuity; it has also removed velocity pull-down and push-up effects on reflector patterns (see for instance solf_2: CDP 300), which are generated by lateral heterogeneity of seismic velocity. The enhanced continuity and higher signal-to-noise ratio provided by the CRS processing significantly contribute to assess the intracrateric reflector configurations and the geometric relationships among different volcanoclastic units.

### Structural interpretation

**Figure 3:**
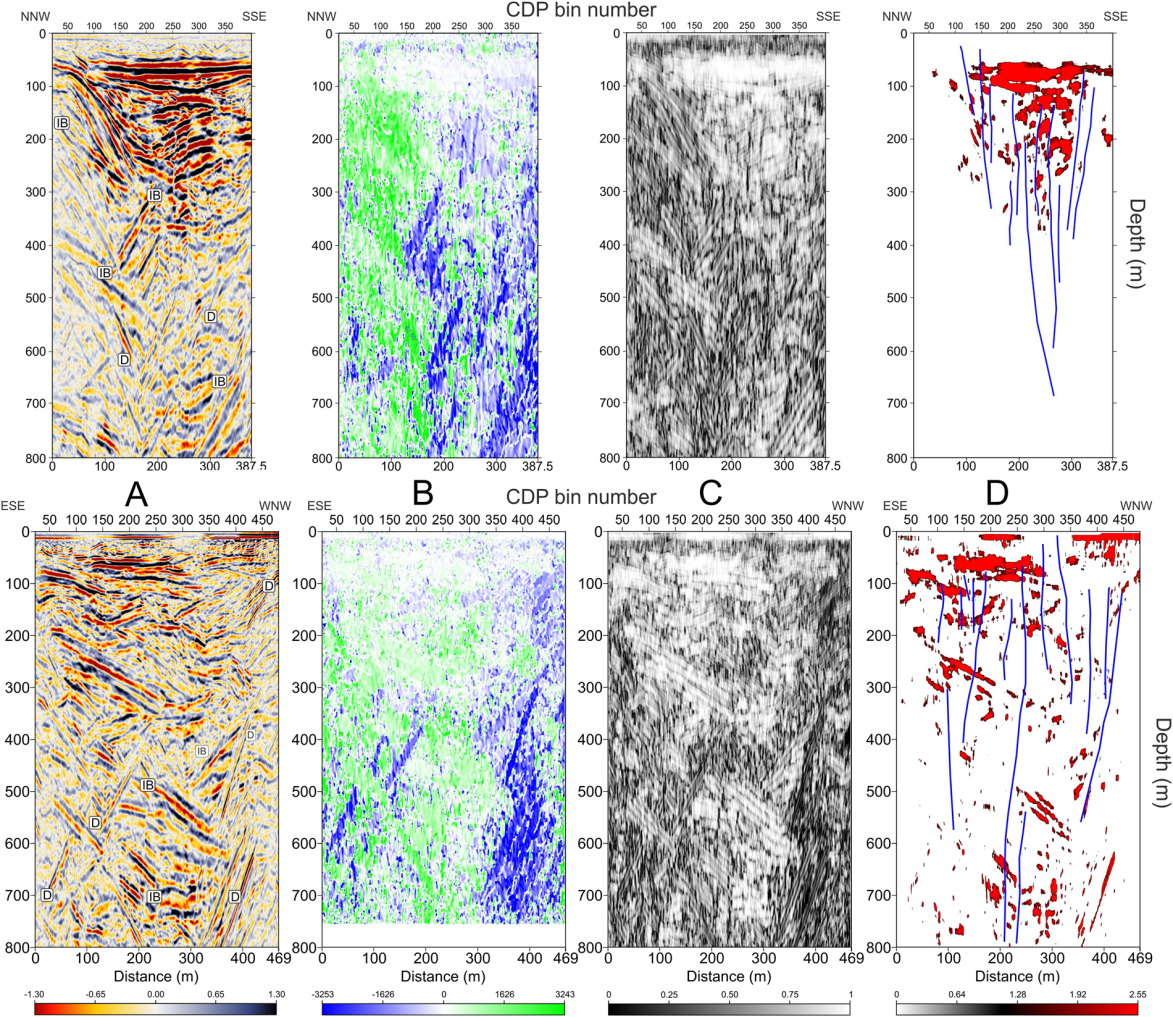
(**A**) Seismic amplitudes of profile solf_1 (top) and solf_2 (bottom) without exaggeration plotted with a gradational red-white-blue palette; positive amplitudes are in black. Label explanation: IB = Intrusive Body; D = Diffraction. (**B**) Dip attribute plotted with a gradational blue-white-green palette, blue colours highlight left-dipping features; (**C**) Similarity attribute plotted using a gradational grey palette; black highlight areas of the profile with the lowest similarity. (**D**) Energy attribute. Red colours highlight areas that have energy greater than 80% of maximum amplitude. Energy values <80% are not plotted. Faults (in blue) are located using both seismic data and dip and similarity attributes.

We used OpendTect Pro for visualization of the depth-converted CRS stacks and for computation of seismic attributes. Pussak *et al.* ^20^ found indeed remarkable improvements in the quality of seismic attributes when the CRS stack was used instead of the conventional CDP stack. In Fig. 3 we compare seismic amplitudes of the two profiles (Fig. 3A) with the dip, similarity and energy attributes (Fig. 3B–D). As discussed in the methods section, the energy attribute enhances changes in seismic impedance; therefore, the plot of Fig. 3D highlights (with a red colour) very high-amplitude horizons and anomalous (i.e. bright) spots within Solfatara that are most likely caused by accumulation of condensed water vapour. In particular, the topmost, broad and flat high-energy area above 100 m deep in Fig. 3D, matches with the highly-continuous low-to-moderate-frequency package of reflectors in Fig. 3A. Most probably, this high-energy area originates at the interface between continuous low-permeability and high-permeability horizons that act as a stratigraphic trap for the uprising hydrothermal fluids.

**Figure 4:**
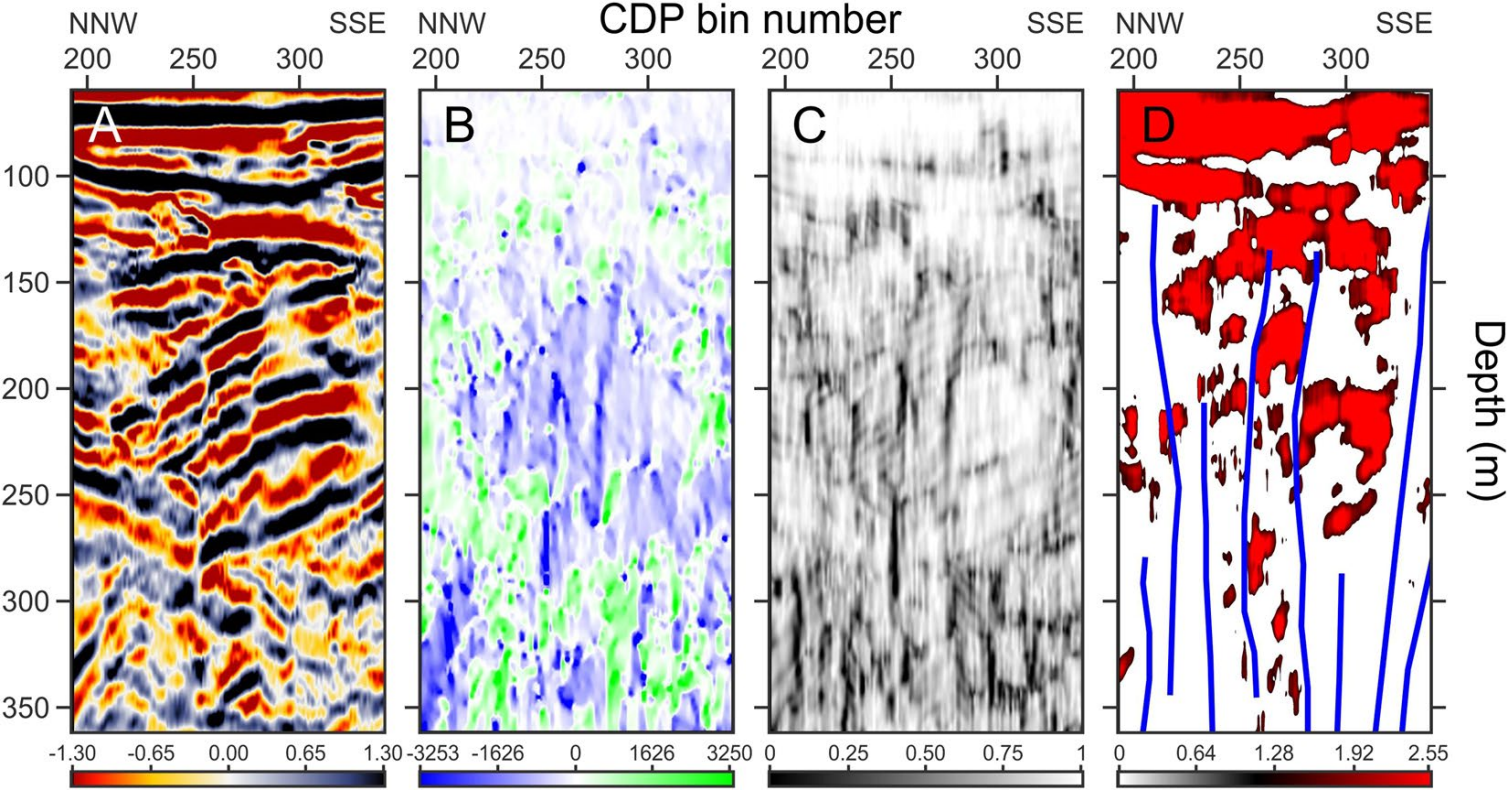
Zoom of seismic amplitudes of profile solf_1 between CDP locations 180–340 and between depths of 80–360 m from datum (i.e. 97 m a.s.l.). Seismic attributes and palettes are as in caption of Fig. 3 and highlight an area affected by subvertical faults. This figure shows the usefulness of plotting seismic attributes for constraining structural interpretation at Solfatara. Faults can be located on seismic data (**A**) as they offset reflectors; they are visible as high, mostly north-dipping segments in (**B**), which match with very low similarity segments in (**C**). Moreover, these faults delimit or terminate below high-energy areas (**D**) created by contact between high-porosity, fluid filled and low-porosity sediments, confirming therefore the crucial role of faults and fractures in controlling the hydrothermal fluid circulation within the subsurface of the crater.

Faults and fractures have been located by matching seismic amplitudes with the information retrieved from dip and similarity attributes. We could thus infer the presence of several sub-vertical faults (in blue in Fig. 3D) disrupting the crater volcanoclastic filling. The usefulness of matching different seismic attributes for constraining structural interpretation at Solfatara can be better perceived in Fig. 4, were we zoom on the seismic attributes of profile solf_1 between CDP locations 180–340 and between depths of 80–360 m. Faults can be easily located on the seismic amplitude plot (A) as they clearly offset reflectors and/or create amplitude reduction/anomaly in the fault zone; at the same time, fault planes are visible as very steep, mostly NNW-dipping (i.e. blue) segments in B, which match with very low-similarity (i.e. black) segments in Fig. 4C.

Isaia *et al*.^19^ point out that faults outcropping at the Solfatara, are related to: (1) volcanic explosions and collapse of the centre of the crater (i.e. ring faults); (2) regional tectonics; and (3) gravity instability of the volcanic rims. Based uniquely on their high dip angle (almost 90°) and on their intracrateric position, we interpret the structures found in our profiles as intracrateric ring faults. In Fig. 3D it can be noticed that these faults delimit or terminate below the above-mentioned water-related bright spots, confirming therefore the crucial role of faults and fractures in controlling the hydrothermal fluid circulation within the subsurface of the crater.

### Stratigraphic interpretation

**Figure 5:**
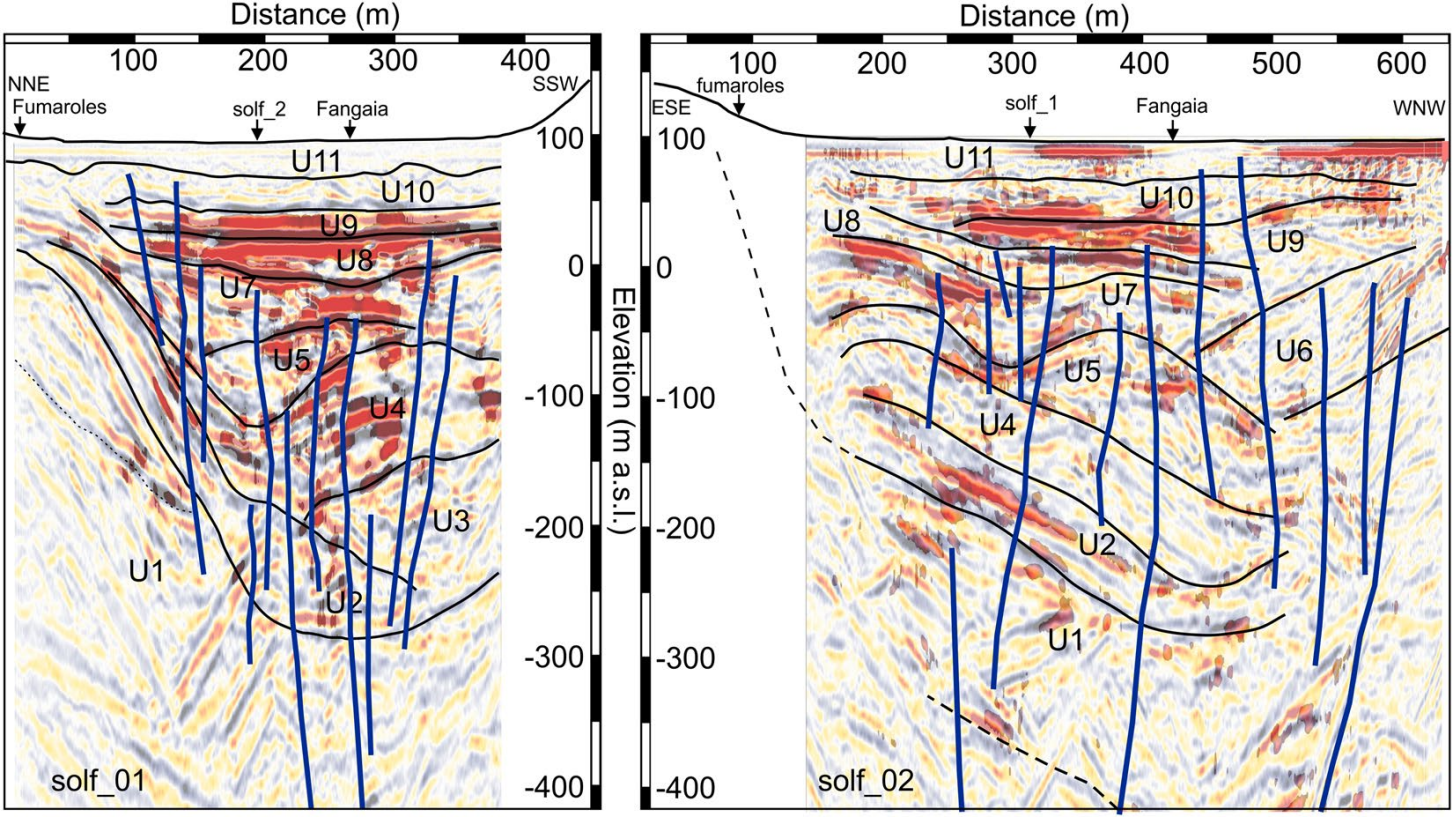
Stratigraphic interpretation scheme for profile solf_1 (left) and solf_2 (right) overlapped to a gradational red-white-blue seismic amplitude plot. Energy values > 80% (red areas) are also overlapped to the interpretation. Both amplitude and energy images are plotted with a 60% transparency. Faults are coloured in blue. For volcanoclastic unit description see text and Table 1. The boundary between U11 and U10 matches with the 2 km/s contour line on the seismic tomograms (supplementary Figs S4 and S5).

We lack well log information to tie to our seismic data, thus our seismic stratigraphic interpretation at Solfatara can be only qualitative and limited to the main unconformities, or changes in seismic patterns, detected on the amplitude plots of the two profiles. We also performed a qualitative analysis of seismic facies of the volcanoclastic deposits from their evident seismic reflection characteristics. In making our interpretation, we adapted the classical seismic sequence analysis schemes^21^ to volcanic settings. Moreover, we used profile solf_1 as a controlling profile, since it has a higher signal-to-noise ratio and it is better oriented with respect to the dip of the volcanoclastic units. Reflectivity and overall data quality is indeed better on solf_1 than on solf_2, mainly due to the higher fold achieved in the field (supplementary Fig. S1) and to the seasonal changes in the near-surface (solf_02 was acquired at the end of a dry summer period). The inferior data quality along solf_2 might also suggest a higher structural complexity of the Solfatara along the WNW-ESE direction^19^, which is also the direction of the main tectonic stress^22^ and of the magmatic source found at 4 km deep by De Siena *et al*.^9^. However, both profiles well portrait the complexity of the volcanic structures filling the crater. Our interpretation presents some minor depth discrepancies at the tie point across solf_1 and solf_2 that are mainly related to the depth conversion, since before this step a good overall match between reflectivity features across the two profiles is evident (supplementary Fig. S2). By integrating the results of seismic tomography at near surface (supplementary Figs S4 and S5) with seismic reflection profiles, we were able to recognize 11 volcanoclastic units, bounded at the top and at the bottom by evident unconformities (see Table 1 and Fig. 5).

**Table 1:** Reflection characteristics of the volcanoclastic units depicted in Fig. 5.

Unit	Upper boundary	Lower boundary	Internal configuration
U11	concordant	concordant	reflection free
U10	concordant	concordant	reflection free
U9	concordant	concordant	parallel
U8	concordant	onlap	parallel
U7	erosional truncation	downlap	oblique progradational
U6	toplap	downlap	oblique progradational/parallel
U5	erosional truncation	onlap	chaotic
U4	erosional truncation	concordant	chaotic to parallel
U3	concordant	downlap	oblique progradational
U2	downlap	concordant	oblique progradational/parallel
U1	erosional truncation	n.a.	reflection free

For seismic facies classification, we used the technique for two-dimensional seismic facies analysis descripted by Ramsayer^21^. Unit U11 has been interpreted to follow the 2000 m/s Vp contour line trend in the tomograms of Figs S4 and S5.

An overall good reflectivity characterizes the crater-filling units (Fig. 3A) and can be used to infer the extent and the shape of the surface carved by the by explosive eruption(s) that deeply cut into the pre-existing country rock (unit U1). The reflective area terminates indeed on an irregular, U-shaped unconformity, clearly recognizable on solf_1 that we interpret as a rupture surface separating a non-reflective pre-eruptive basement (U1) from the reflective volcanoclastic units (U2–11). This surface is also well visible on solf_2 as a series of low-frequency reflective events that define an overall concave surface. On the depth-converted CRS images, the contact between U1/U2 reaches a maximum depth of ~400 m below the crater surface, which is approximately 2/3 of the crater diameter (i.e. about 600–700 m).

The overall non-reflective basement (U1) hosts a set of high-amplitude, low-frequency planar events with opposite dips, found and at depths from 200 to 800 m (“IB” in Fig. 3). Their dip angles range between 10–40°, preferentially to the SSE on solf_1 and to the WNW on solf_2. These anomalous events are similar in their characteristics to features observed in seismic profiles across the Vøring and Møre basins, offshore Norway^23^ and in Taranaki Basin, New Zealand^24^ and interpreted as intrusive bodies (i.e. sills and dykes). Their positive polarity and high amplitude is due to the high difference in acoustic impedance between them and their volcanoclastic counterparts. Other sets of high-amplitude, high-frequency events (labelled as “D” in Fig. 3) showing both positive and negative polarity and dipping with higher angles (60–68°), can also be noticed within seismic unit U1. These last events are most probably noise, i.e. diffractions whose strength has been reinforced by the CRS processing.

Moving within the crater-filling units (U2-U11) we notice that reflectivity becomes higher than U1 but it is not constant. For instance, units U5-U6 are characterized by more transparent reflectivity with respect to U4, U8 and U9. This change in seismic character indicates changes in composition and homogeneity of the volcanoclastic units filling the crater. Moreover, most crater-filling units have complex reflection configurations. Stratigraphic complexity seems indeed inversely correlated with stratigraphic position (i.e. age) of the crater-filling material. Units U8 to U11 are almost sub horizontal with mostly parallel or sub-parallel reflection configurations and concordant upper and lower boundaries, while, older units (i.e. U2-U7) seem to show erosional truncation boundaries and oblique to sigmoid reflection configurations, similar to those found in progradational sedimentary environments.

## Discussion

CRS processing was crucial in improving the signal-to-noise ratio and the reflector continuity of the two profiles acquired within the Solfatara crater. The CRS method leads to considerable small-scale improvements even in the near-surface section, with a better identification of faults, as the reflection continuity is generally increased, but without smoothing or removing the reflector discontinuities^25^. The improved signal-to noise ratio and continuity achieved by CRS processing allowed us to fully exploit the high-resolution information embedded in the two 2D seismic surveys and to gain clear and detailed images of the volcanoclastic filling of the crater. Thanks to their overall good quality and high-resolution, our results can provide volcanologists with a solid framework to constrain the near-surface geological interpretation of this very complex and dynamic environment, and can improve our understanding of the recent temporal changes affecting this sector of the CF Caldera.

The primary goal of this survey was indeed the location of the intracrateric faults and fractures. Our profiles clearly show several steep and segmented intracrateric collapse faults, generally with normal kinematics and dipping toward the crater centre. Fault zones are imaged on our seismic data as sudden amplitude drops that generate narrow low-similarity and high-dip attributes. These seismically imaged fault zones are narrow, with maximum widths of 10 m, while measured fault offsets are generally small (i.e. not more than 30 m). Fault zones at Solfatara are very likely to be filled by fluids degassed by a magmatic source and this causes the small-scale amplitude anomalies seen along the fault planes and well preserved by CRS processing^26^. Our seismic profiles show a diverse spatial distribution of fractures and faults. Most of these structures terminate below units U7-U8 while only a few of them, mainly located on the NNE and WNW rims, seem to affect the surface units (U9-U11). Probably these last faults are also the main degassing pathways feeding the Fangaia and the fumaroles of Solfatara (Fig. 5). This different vertical distribution of faults can reflect a different age of these structures, or, more simply, can be the result of lithological changes occurring in upper part of the crater, where unconsolidated or poorly consolidated materials are more abundant. Seismic stratigraphic features of U9-U11, matched with surface geological mapping^19^ and with previous geophysical surveys^11–14^ suggest indeed that these units most probably consist of packages of loose volcanoclastic deposits with different degree of permeability, within which fault offsets, and their associated velocity anomalies, especially if not large, might not be well preserved as in the more consolidated material below. We also note that most of the high-amplitude bright spots are located within units U8-U9 and in part U10, just above the upper fault terminations (Figs 3 and 5). As discussed in the previous section, these bright spots might represent the seismic image of unconsolidated high-permeability horizons, bounded above by low-permeability horizons and saturated by fluids uprising along the fault planes (Fig. 3D).

Another important goal of this survey was indeed the assessment of the structural and volcanological setting of the Solfatara crater. At first glimpse, the two profiles allow estimating the volume and the overall shape of the crater carved within the non-reflective pre-eruptive basement (U1) and filled with reflective volcanoclastic material. Additionally, by examining in detail the internal reflective configuration of the filling units and their bounding unconformities, and intersecting these seismic stratigraphic features with the findings of Isaia *et al*.^19^, we can interpret the shallow units (U8-U11) as tephra ejected by Solfatara and nearby eruptive centres (Olibano, Santa Maria delle Grazie, Mt. S. Angelo, Astroni, Accademia and Agnano-Monte Spina). This hypothesis is also supported by the clear onlap reflection configurations found within some of these units (i.e. U8-U9). Onlap relationships are indeed compatible with deposition of falling volcanic material that will drape the previous morphology. The deeper units (U2-U6) show instead pronounced dips, chaotic and sigmoid to oblique reflection configuration. Some depositional patterns show clear progradations above erosional surfaces (i.e. downlap of U7 above U5 in Fig. 5). These pieces of evidence may be indicative of an emplacement by pyroclastic density currents and minor fallout and/or of sin- or post-depositional slumps (U2-U4) triggered by the large dips of the crater rims and by the collapse of the crater.

Finally, our seismic profiles and their interpretations (Figs 3 and 5) provide some important evidence in support of Isaia *et al.* ^19^ who interpret the shallow structure of Solfatara crater as a maar (see White and Ross^27^, and references therein). We find indeed: (1) a crater that cuts deep into pre-existing non-reflective country rock U1; (2) abrupt sub-vertical contacts between the crater-filling units and the country rock; (3) evidence of intracrateric (collapse) ring faults; (4) evidence of intracrateric collapse of early (i.e. U2-U4) volcanic units; (5) an evident seismic character differentiation between the upper part of the crater (U2-U11), which is characterized by well-defined beds, and the lower part (U1) in which there is a week evidence of layering (Fig. 3A). However, our profiles do not show seismic features that may suggest the presence of a central chimney which is often found within the diatreme structure underlying the upper maar^19,27^.

## Methods

We collected the two 2D seismic profiles within the crater (Fig. 1B,C) along almost orthogonal NNE-SSW and WNW-ESE directions, which are also roughly related to the main structural trends found at the Solfatara^19,27,28^. The profiles intersect each other near the centre of the crater. The NNE-trending profile, named as solf_1, is 430 m long, while WNW-trending profile, solf_2, is 478 m long. We used the differential GPS technique to acquire the position of all geophone and vibration points; horizontal and vertical position errors are 0.02 m and 0.1 m, respectively. The change in the elevation along the profiles is 9.1 m for solf_1 and 5.3 m for solf_2 (supplementary Fig. S1), with a maximum elevation of 105.4 m at the eastern end of the profile solf_1, a minimum of 96.3 m occurring at metric position 236 along solf_1, and an average elevation of 98.3 m. The most significant elevation changes occur at both edges of the profile solf_1 toward the crater rims; elevation variations within the crater are less than 5 m for solf_1 whereas the topographic pattern is more articulated for profile solf_2. Hard logistic conditions in the crater site and very hot surface conditions with temperature above 80 °C and a strong oxidation environment did not allow to extend the seismic surveys further toward the ridges of the crater.

As a seismic source, we used a single 6400 kg IVI-MINIVIB® vibroseis truck, which delivers a maximum theoretical peak force of ~27 kN at each sweep. A nominal source spacing of 4 m was used, with vibration points located halfway between geophones. The choice of the acquisition parameters, allowed us to acquire data with an overall very dense CDP spacing (1 m) and high fold. However, some source gaps are present, especially along solf_2 (supplementary Fig. S1), as this profile intersects areas that were either unsafe, too hot or characterized by rough surface conditions to operate the vibrator. We acquired 117 vibration points along profile solf_1 and 75 vibration points along solf_2. This lower number of vibration points influenced the total fold and consequently the signal-to-noise ratio for solf_2 is consistently lower than for solf_1 (supplementary Fig. S1). The maximum fold for profile solf_1 exceeds 120% while it is limited to 76% for solf_2. At each vibration point, two 15 s long, 5–150 Hz sweeps were stacked and recorded respectively by a 216-channel (i.e. solf_1) and 240-channel (solf_2) array, with spacing between the sensors of 2 m. The source-receiver configuration allowed for sampling a wide range of offsets, from a minimum of 0.5 m to a maximum of 453 m.

The workflow used for processing the two reflection profiles is detailed in supplementary Fig. S3. An exhaustive description of the processing phase is beyond the scope of this paper; therefore, we limit the description to the major stages. Details can be found in Yilmaz^29^ and references therein. Signal-to-noise ratio in our recorded data is degraded by the high level of ambient (i.e. activity of fumaroles) and anthropic noise, especially at large offsets (see the shot gathers of Fig. S1). To improve the signal-to-noise ratio and the temporal resolution, surface-wave removal algorithms and spiking deconvolution were applied. The pre-processed shot gathers were corrected for refraction statics and referenced to a common topographic level at 97 m a.s.l. Afterwards, semblance- and tomography-based methods were used to define a reliable velocity model (V_RMS_) for the CDP ensemble stack. However, the lack of clear horizons on the common midpoints strongly limited the success of the semblance in providing a good estimate of stacking velocities. This is the main reason of the poor quality of the CDP stacks, as shown in Fig. 2. In developing reflection stacks, we applied Normal Moveout (NMO) and Dip Moveout (DMO) corrections, residual statics and finally CDP stacking.

To improve signal-to-noise ratio we also processed our data with the CRS method^17,18^. The CRS stacking does not directly require a velocity model, but it uses instead three parameters, namely the CRS attributes that have to be estimated. These attributes describe the reflected wavefront associated with two eigenwaves^26^. Several strategies have been developed in the recent years to search for the three CRS attributes^30,31^. For our data we adopted a three-step algorithm, which searches for the CRS parameters using the NMO-corrected and stacked section by assuming that it approximates a zero-offset (Z_O_) section, and performs 1D optimization steps for each of the three CRS attributes^18,30,32^. The CRS stack operator does not depend on a velocity macro-model; however, the velocity information from CDP processing can be used as a guide function in the CRS search strategy^18^. The algorithm searches for the dips of emerging zero-offset wavefronts in the zero-offset domain, using the CDP stacks. Afterwards, an estimate of near surface velocity should also be provided, and it is used together with the dip and V_NMO_ information to search for the other attributes of the CRS stacking operator.

The Z_O_ aperture is the most important parameter to test in high-resolution applications. It defines a CDP-domain radius within which the data will be stacked. In choosing the Z_O_ aperture there is a trade-off between signal-to-noise enhancement and lateral resolution^17^. A careful preliminary analysis of shot gathers is therefore essential to choose Z_O_ apertures to optimize this trade-off. We tested a wide range of Z_O_ apertures and the best compromise between signal-to-noise ratio and seismic resolution was provided by an aperture progressively increasing with recording time, from 25 m at 0 s to 100 m at 1 s. These values approximatively match the size of the first Fresnel zone along the profiles.

To provide a background model for depth conversion of both CDP and CRS stacks, we used the smoothed final stacking velocity (V_RMS_) model obtained during the CDP processing. This model provided velocity estimates for the deeper part of the profile and it was integrated in the shallow part with the tomographic results discussed in supplementary material.

We used OpendTect Pro to compute Energy, Similarity and Dip-Steering attributes on the depth converted CRS stacks. The Energy attribute computed in OpendTect is the squared sum of the sample values in a time-gate (in our case 5 m) divided by the number of samples in the gate. The Energy attribute is therefore a measure of reflectivity strength within the chosen time-gate.

OpendTect Similarity is a multi-trace attribute that returns trace-to-trace similarity properties. First, the algorithm searches for the direction of best match between adjacent traces at every dip angle. Afterwards, using the best match dip angle, the similarity between adjacent traces is computed by considering the trace segments as vectors in a high-dimensional, synthetic space such that the proximity between two points in the coordinate space is correlated with the similarity between the trace segments that the points represent. In such a space, similarity is then defined as one minus the Euclidean distance between the vectors, normalized by the vector length. The trace segment is defined by the time-gate in ms and the positions are specified in relative coordinates. In the case of 2D data the trace positions are defined by the trace step-out only. An extension parameter determines how many trace pairs are used in the computation. A computed similarity of 1 means that the trace segments are identical in waveform and amplitude. A similarity of 0 means that they are completely dissimilar.

Finally, the dip-steering is a process that produces a steering cube, i.e., a volume that stores information about the seismic dip of coherent events at every sample position. The algorithm computes the dip from the gradient of the unwrapped instantaneous phase; the process is fast but produces some high-frequency noise that is removed by smoothing the results.

### Data availability

The datasets analysed during the current study are available from the corresponding author on reasonable request.

## Electronic supplementary material

Supplementary Material
