# Supplementary material for: The shallow structure of Solfatara Volcano, Italy, revealed by dense, wide-aperture seismic profiling
Source: Sci Rep. 2017 Dec 12;7:17386. doi: 10.1038/s41598-017-17589-3 (PMC5727181; doi:10.1038/s41598-017-17589-3)
Supplement: Supplementary file 1 — Supplementary Material [file 41598_2017_17589_MOESM1_ESM.pdf]

Supplementary Material of

The shallow structure of Solfatara Volcano, Italy revealed by dense, wide-aperture seismic profiling

Pier Paolo G. Bruno[1,*] Stefano Maraio[2] and Gaetano Festa[3]

[1] Khalifa University of Science and Technology, Petroleum Institute, P.O. Box 2533, Abu Dhabi, United Arab Emirates

[*] pbruno@pi.ac.ae

[2] Università degli Studi di Siena, Centro di GeoTecnologie, S. Giovanni Valdarno, Italy

[3] Università degli studi di Napoli Federico II, Dipartimento di Fisica “Ettore Pancini”, Naples, Italy

Seismic data acquisition.

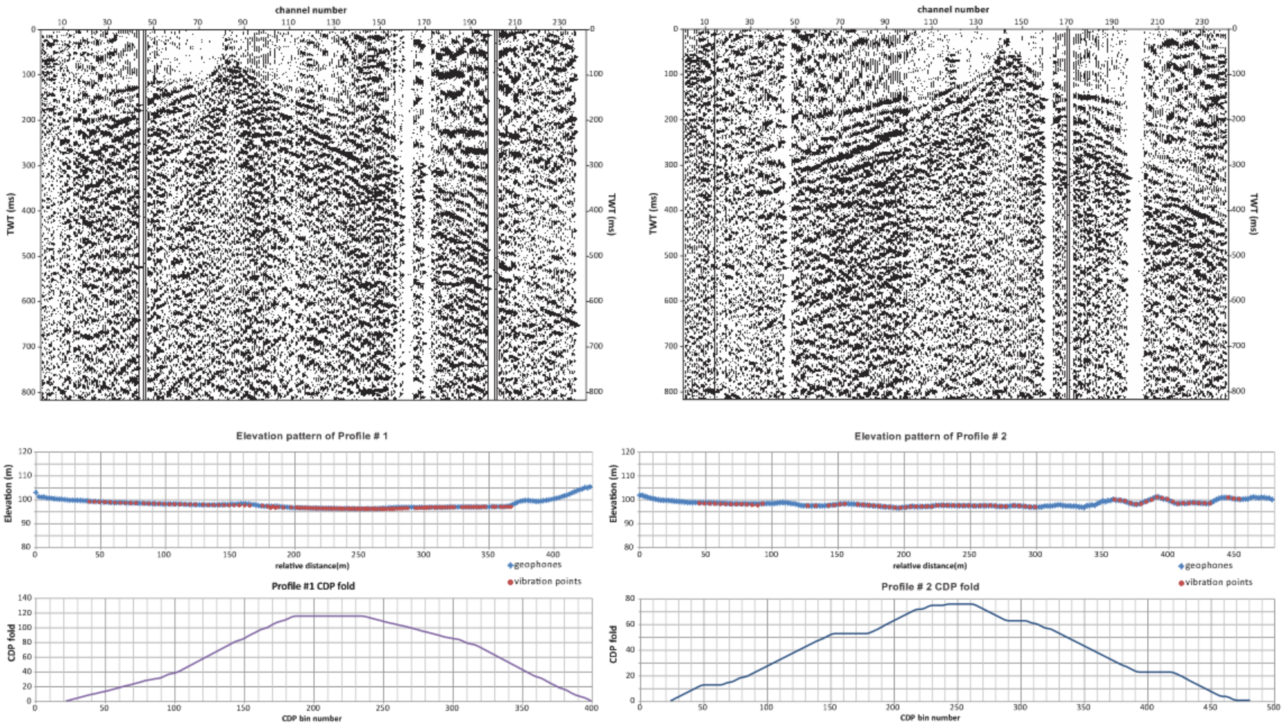

Figure S1. Top images: two representative seismic shot gathers recorded along profiles solf_1 (left) and profile solf_2 (right). Some basic processing, i.e. band-pass filtering and amplitude recovery, is applied to the data to improve signal-to-noise ratio. Middle images: topographic pattern of geophones (blue dots) and vibration points (red dots) for both profiles. Notice a more rugged topographic pattern for profile solf_2 (right). Bottom images CMP fold map for profiles solf_01 and solf_02. Lower fold for solf_2 is related to a larger number of acquisition gaps related with the rugged topography.

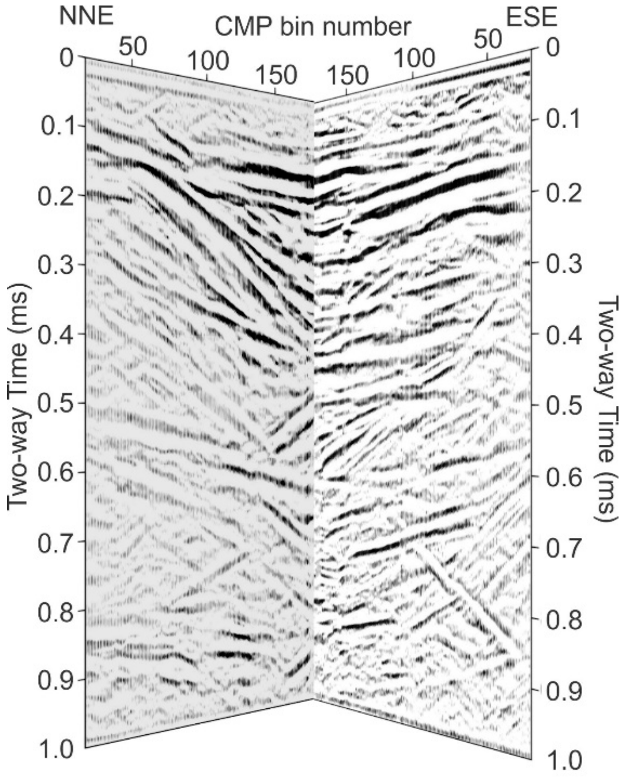

Figure S2. NE view of the intersection between profile solf_1 (left) with profile solf_2 (right). It can be noticed a good overall match between depth, amplitude and frequency of the reflectivity sequence along the two orthogonal profiles**.**

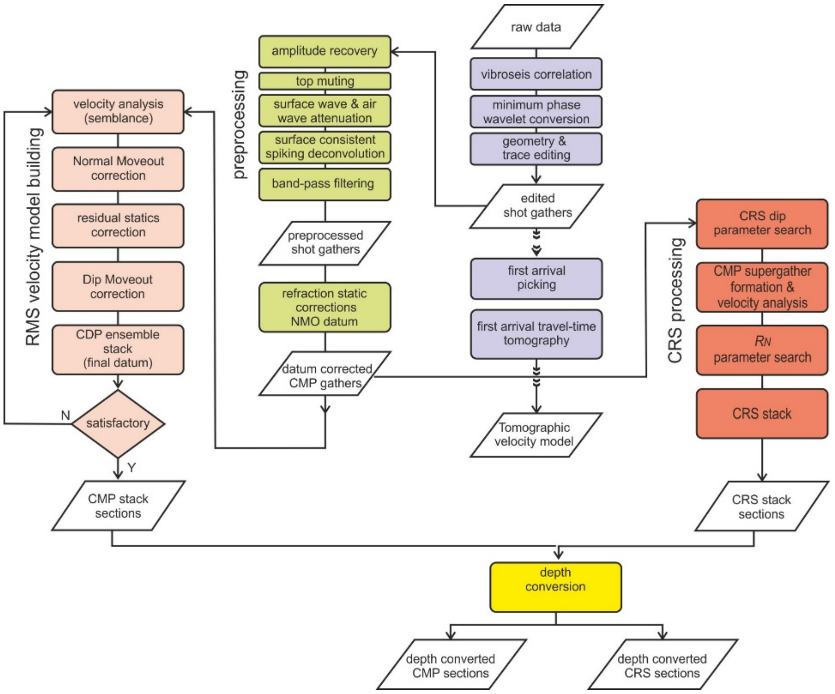

Figure S3. Data processing flow. The upper left side of the diagram illustrates the algorithms (green) applied to pre-process all data before differentiating the processing steps. The lower left side of the diagram shows then a conventional CDP processing scheme (pink) which outputted our CMP stack sections. The upper central side of the diagram (blue) illustrates the processing steps applied to correlate the raw field data and prepare them for first arrival picking and for tomographic inversion. In the central part of the diagram (red) the CMP stack sections and the datum-corrected CMP gathers are used as input to CRS processing. Both CMP and CRS stack sections have been depth converted.

Seismic tomography.
After vibrosesis correlation and minimum phase conversion, we picked the first-arrival P-wave phases on the common-shot gathers. The arrival times were then inverted using travel-time tomography[1]. The use of dense, wide-aperture arrays allowed us to record highly redundant, global-offset first arrivals that were further checked for consistency using the reciprocity rules of Ackermann et al[2]. Theoretically (i.e. in noise free seismograms), the indetermination on first arrival picking is around 1/8 of the dominant period (in this case ~ 3 ms). In some parts of the profiles and especially at large offsets (see for example Fig. **S1**), decrease in the signal-to-noise ratio and high heterogeneity in the subsurface increased the picking uncertainty up to almost one order of magnitude larger than the theoretical value. In many cases the picking of the large offset head-waves was thus impossible to obtain.

Theoretical travel times were obtained via a ray-tracing method, based on the Huygens principle[3] using a preliminary P-wave velocity provided by classical refraction methods of interpretation. We discretized our a priori model on a computation matrix with size of 107x24 nodes for solf_01 and 120x24 nodes for solf_02. All nodes are regularly spaced of 4 m along the horizontal direction and of 4.5 m along the vertical direction. The grid covers the entire length of the profiles, from topographic surface down to a depth of ~100 m. Residuals between observed and theoretical travel times were minimized upgrading the preliminary velocity model with an iterative tomographic approach based on the SIRT image reconstruction technique[4]. The final residual root-mean-square (RMS) errors on traveltimes are 8.3 ms for solf_01, and 10.3 ms for solf_02, this latter profile being affected by lower signal-to-noise ratio than solf_01.
Resolution of tomographic images is assessed by representing ray density plots and by computing a posteriori checkerboard tests[5]. Ray density plots provide a qualitative measure of the resolving power of refraction tomographic results, showing the number of seismic rays that cross each cell of the model. The larger the number of ray coverage per cell the better the resolving power. Checkerboard tests provide instead a quantitative estimation of model resolution[6]. We perturbed the final tomographic velocity models using a synthetic pattern similar to a checkerboard. We chose cell dimensions of 40mx20m and perturbation values of ±10% of the cell P-wave velocity (Figs. **S4** and **S5**). The degree of reconstruction of the perturbed pattern provided by the tomographic algorithm is used to assess the reliability of the details provided in the tomographic model[6].
The analysis of the checkerboard tests shows an achieved depth penetration of only 40-50 m, about 1/10 of maximum offset. This limited penetration is mainly due to the limited number of head-wave pickings at large offsets.

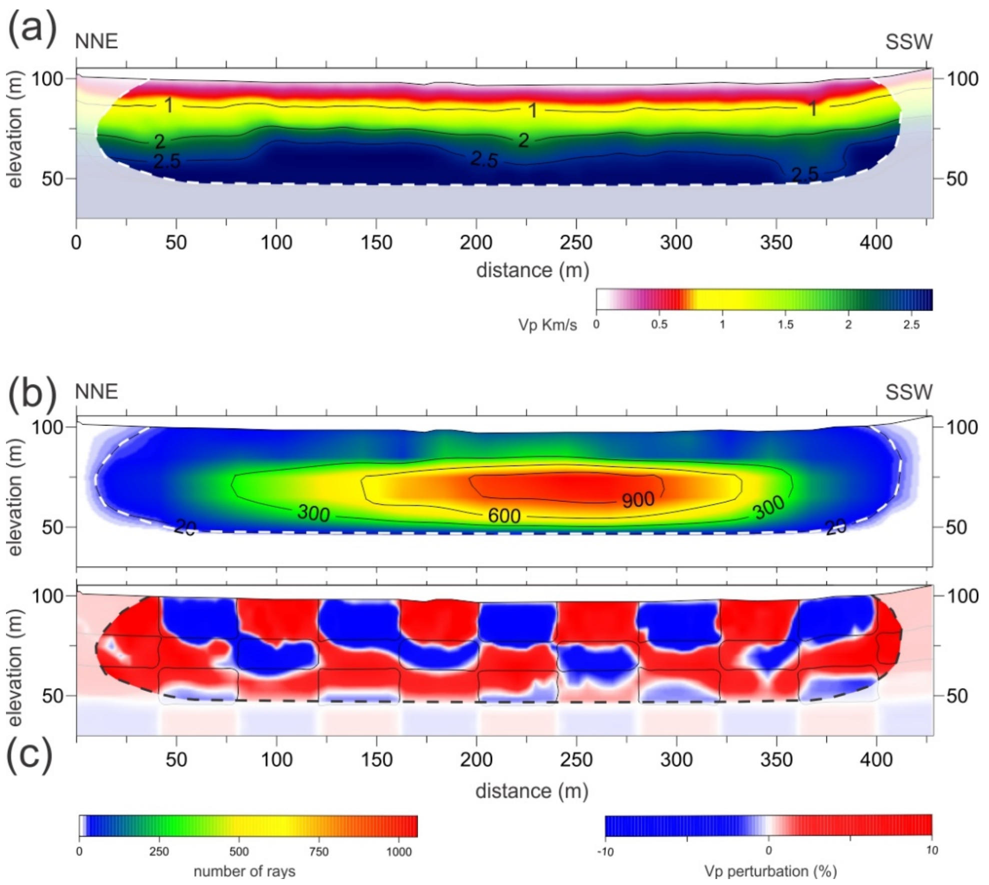

Figure S4. a): results of Vp refraction tomography along profile Solf_1; b): number of rays per cell; c): perturbation pattern retrieved after the “a posteriori” checkerboard resolution tests. The RMS traveltime error for the final model is 4.1 ms. The input perturbation pattern has values of ± 10 m/s in the cell with horizontal size 40 m and vertical size 20 m. Resolution depth is evaluated according to the retrieved pattern and the value of velocity perturbations, along with the ray coverage. Vertical and horizontal scales are equal.

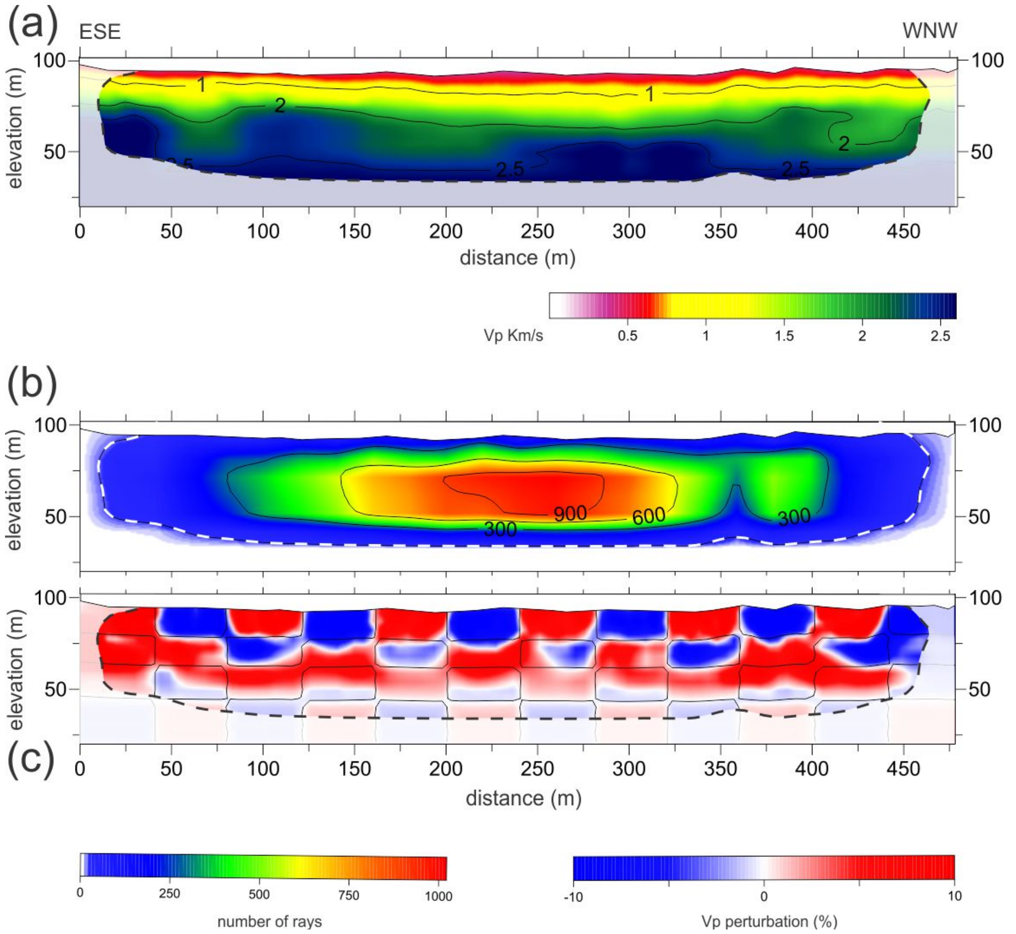

Figure S5. a): results of Vp refraction tomography along profile Solf_2; b): number of rays per cell; c): perturbation pattern retrieved after the “a posteriori” checkerboard resolution tests. The RMS traveltime error for the final model is 4.1 ms. The input perturbation pattern has values of ± 10 m/s in the cell with horizontal size 40 m and vertical size 20 m. Resolution depth is evaluated according to the retrieved pattern and the value of velocity perturbations, along with the ray coverage. Vertical and horizontal scales are equal.

Comparison with previous surveys.

In this part we compare our tomography results shown in Fig. **S4** and **S5** with the high-resolution P-wave tomography of the first 35 m of the central part of the crater obtained in the same period by de Landro *et al.*,[7] using the active-source data from the 3D RICEN array (Fig.1b). The comparison with older high-resolution tomographic images of the crater[8] is less useful due to the large variations that occurred in the near surface of this extremely dynamic environment in the recent years. de Landro *et al.*,[7] integrated their tomography with the resistivity, temperature and CO_2 flux measurements, and interpreted the following near-surface characteristics: 1) a depth-dependent P-wave velocity layer down to 14 m, with Vp < 700 m/s typical of poorly-consolidated tephra and affected by CO2 degassing; 2) an intermediate layer, deepening towards the mineralized liquid-saturated area (Fangaia), interpreted as permeable deposits saturated with condensed water; 3) a deep, confined high velocity anomaly that de Landro *et al.*,[7] associate to a shallow CO_2 reservoir.

Overall, the model of Landro *et al.*,[7] and our tomographic profiles are characterized by similar velocities in the overlapping depth range (0-35 m). However, a detailed comparison of tomographic features is hampered by the different orientation of our 2D profiles with respect to the array used by Landro *et al.*,[7]. This is evident in Fig. **S6**, that shows our profiles intersecting the high-velocity anomaly of de Landro *et al.*,[7] in peripheral areas where the anomaly has widths of 10-30 m and thicknesses of 2-3 meters, clearly beyond the resolution of our models (see checkerboard tests in our Figures **S4** and **S5**). Moreover, since the model of Landro *et al.*,[7] has a spatial resolution 10mx10mx5m, the projection of their anomaly on the WNW-trending profile solf_2 has an uncertainty comparable to the expected size of the anomaly.

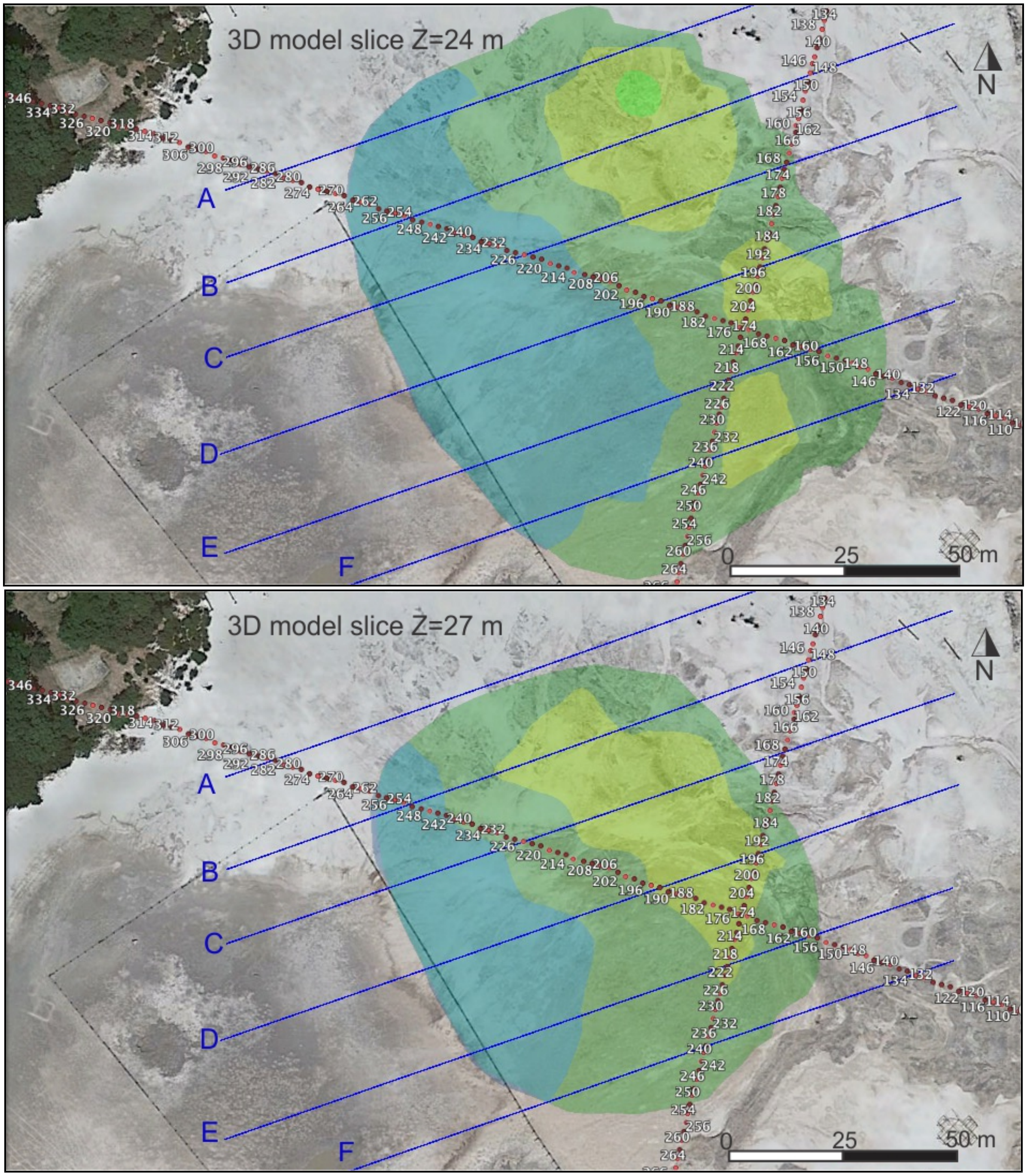

Figure S6: 2D image maps of Solfatara (© 2017 Google), showing the spatial relationships between our 2D tomographic profiles solf_1 and solf_2 (in red with labels indicating the offset) with the 2D vertical slices extracted from the tomographic P-wave volume of de Landro et al. (A-F in blue). On the top and bottom images are also plotted two depth slices from de Landro *et al.*,[7] (respectively Z=24 m and Z=27 m), across the high P-wave anomaly (in yellow; Vp >1500 m/s).

Therefore, because of the different orientation and spatial resolution of the two RICEN experiments, our surveys are unable to confirm the exact location and shape of the high-velocity anomaly inferred by de Landro *et al.*,[7]. For instance, in the tomogram of Fig. **S5** the projected position of such anomaly on profile solf_2 is between metric positions 165-195, just above an opposite (negative) and wider velocity anomaly. However, we notice two small positive anomalies to the left (meters 10-50 and 80-150) and a wider one to the right (meters 225-340) of the predicted position of the anomaly of de Landro *et al.*,[7]. In particular, the wider anomaly on the right can be the same as the gas reservoir of de Landro *et al.*,[7] or its root. These relatively deeper anomalies (of which our tomography cannot characterize their precise shape because we are at the limit of resolution) can be interpreted, in agreement with de

Landro *et al.*,[7] as regions of storage of gas, which upraises along the faults and fractures identified by the seismic reflection survey.

Both our profiles and those of de Landro *et al.*[7] do not show any clear evidence of surface faulting. This lack of evidence is in agreement with the results of the seismic reflection survey that found most of the intracrateric faults to remain largely confined to depths greater than 90-100 m. The few faults that do seem to reach the surface probably generate only subtle geophysical signals that are below the resolution power of the tomographic images. Both 2D and 3D[7] tomographic models are probably be too smooth to well represent almost vertical features.

References

1. Iyer, H. M., and Hirahara, K. (Eds.). Seismic tomography: Theory and practice. *Springer Science & Business Media* (1993).
2. Ackermann, H. D., Pankratz, L. W., and Dansereau, D. Resolution of ambiguities of seismic refraction traveltime curves, *Geophysics*, **51**(2), 223-235 (1986).
3. Hayashi, K., and Takahashi, T. High resolution seismic refraction method using surface and borehole data for site characterization of rocks, *International Journal of Rock Mechanics and Mining Sciences*, **38**(6), 807-813 (2001).
4. Gilbert, P., Iterative methods for the three-dimensional reconstruction of an object from projections. *Journal of theoretical biolog*y, **36**(1), 105-117(1972).
5. Hearn, T. M., and Ni, J. F., Pn velocities beneath continental collision zones: the Turkish-Iranian Plateau, *Geophysical Journal International,* **117**(2), 273-283 (1994).
6. Rawlinson, N., Fichtner, A., Sambridge, M., and Young, M.K., Chapter One - Seismic Tomography and the Assessment of Uncertainty. *Advances in Geophysics, Elsevier*, **55**, 1-76 (2014).
7. de Landro et al. 3D ultra-high resolution seismic imaging of shallow Solfatara crater in Campi Flegrei (Italy): New insights on deep hydrothermal fluid circulation processes. *Scientific Reports*, **7** (2017).
8. Bruno, P. P. G. et al. Geophysical and hydrogeological experiments from a shallow hydrothermal system at Solfatara Volcano, Campi Flegrei, Italy: Response to caldera unrest. *Journal of Geophysical Research: Solid Earth*, **112**(B6) (2007).
